# Development and validation of a framework and scale for primary and secondary school teachers’ data-artificial intelligent competence

**DOI:** 10.3389/fpsyg.2025.1756893

**Published:** 2026-01-14

**Authors:** Jianli Fan, Haibin Wang, Xiulin Gu

**Affiliations:** 1 School of Educational Sciences, Huangshan University, Huangshan, China; 2 School of Education, Soochow University, Suzhou, China

**Keywords:** artificial intelligence, data-artificial intelligent competence, framework construction, primary and secondary school teachers, scale validation

## Abstract

**Background:**

As generative AI and other technologies reshape the educational ecosystem, teachers’ data - artificial intelligent competency (DAIC) has become the core bridge connecting technological innovation with teaching practice.

**Methods:**

This study employs a mixed-methods approach to construct a DAIC framework for K-12 teachers and develop a standardized measurement scale for validation. The framework dimensions were first established through thematic mining of 33,800 teacher competency demand texts, combined with two rounds of Delphi consultations involving 28 education experts. Subsequently, a cross-sectional survey was conducted using stratified random sampling. Exploratory factor analysis was performed on 215 pre-survey data points, while confirmatory factor analysis and reliability/validity testing were applied to 2,052 formal survey responses.

**Results:**

The teacher DAIC framework comprises five core dimensions of Data Literacy Awareness and Beliefs, Data Literacy Knowledge and Skills, Higher-Order Data Literacy Thinking, Data Literacy Teaching/Learning Application, and Related Personality Traits. The 25-item scale demonstrates strong internal consistency and construct validity (Cronbach’s α=0.983, χ^2^/df=3.11, CFI=0.938, TLI=0.931, RMSEA=0.046, SRMR=0.040).

**Conclusion:**

This study integrates relevant theories to reveal the intrinsic logic of merging data literacy with AI literacy, overcoming the fragmented limitations of existing research that analyzes them separately. Besides, it supplements localized evidence of teachers’ DAIC within the Chinese context, specifically addressing cultural adaptability and low-resource environment suitability issues in international frameworks. The developed scales and low-threshold application solutions adapted to urban-rural disparities provide actionable pathways for teacher professional development in resource-constrained regions. This framework and scales balance theoretical rigor with practical applicability, offering scientific tools for differentiated teacher training and regional educational informatization assessment. They also provide reference for localizing international teacher digital literacy frameworks, thereby advancing equitable educational digitalization.

## 1 Introduction

The global landscape of basic education is undergoing unprecedented transformation driven by the convergence of generative artificial intelligence (GenAI), learning analytics, and immersive technologies (Lee and Low, 2024). The UNESCO 2024 Global Education Digital Transformation Report explicitly states that education has shifted from technology-enabled teaching to data and AI-driven teaching. This transition requires educators to transcend traditional digital skills and cultivate a composite competency integrating data literacy, AI collaboration capabilities, and ethical judgment, referred to as Teachers’ Data-Artificial Intelligence Competence (DAIC) (Ji et al., 2024). This competency is not only central to implementing the OECD’s 2030 Framework for Effective Learning Environments but also a critical lever for narrowing the global digital divide in teacher capacity building (Zigama, 2025). The balanced development of teacher competencies in the digital-AI era has become a vital issue in global education governance.

From the perspectives of global practice demands and academic research contexts, recent international initiatives have highlighted the urgency of defining DAIC while also revealing practical challenges and research limitations. UNESCO’s AI Competency Framework for Teachers proposes that “teachers must become proactive coordinators of the digital-AI learning ecosystem,” with human-machine collaborative decision-making and data-driven instructional design emerging as core requirements (Okada et al., 2025). Meanwhile, the proliferation of adaptive learning platforms like Class Dojo Insights and classroom analytics tools demands that teachers possess the ability to interpret real-time data, validate AI recommendations, and mitigate ethical risks. However, significant regional disparities in DAIC proficiency exist. Evidence from TALIS indicates that only 35% of teachers in low- and middle-income countries (LMICs) confidently use AI in teaching, compared to 68% in high-income countries (Jerrim and Sims, 2022). The absence of a globally applicable assessment framework leads to fragmented training. For instance, Europe’s Dig Comp Edu emphasizes digital skills while downplaying AI ethics (Caena and Redecker, 2019). It is evident that existing research suffers from numerous shortcomings. For instance, data literacy-oriented research emphasizes data application while neglecting AI collaboration; AI literacy-focused studies prioritize AI cognition but marginalize data literacy; and comprehensive digital competence frameworks cover broad domains yet lack detailed design for intelligent technologies. These shortcomings share a common issue of insufficient context sensitivity. Moreover, most research is developed based on high-income country contexts, disregarding resource constraints in LMICs and disconnecting from the practical needs of teachers in settings like rural schools.

To address these gaps, this study integrates existing findings on data literacy, AI literacy, and digital competence to construct a novel DAIC framework. This framework aims to fill existing research deficiencies while incorporating contextual adaptation considerations. Employing a mixed-methods design, the study captures practical needs through extensive surveys of frontline teachers’ competency requirements. Combining these insights with the Delphi method for framework optimization, it ultimately develops and validates a cross-context applicable teachers’ DAIC. The research centers on two core questions:

Question 1: How should the core dimensions and constituent elements of teachers' DAIC be defined to achieve a balance between global standards and contextual adaptability?

Question 2: How can a psychometrically sound teachers' DAIC scale be developed and validated to accurately assess teachers' competency levels across diverse educational contexts?

## 2 Literature review

### 2.1 Definition of DAIC for primary and secondary school teachers

Based on the core definition of the ability of numerical intelligence in educational scenes in the OECD (2025) Framework for Teachers’ Artificial Intelligence, combined with the professional particularity of primary and secondary school teachers, this study holds that the teachers’ DAIC refers to the ability to collect, analyze and interpret teaching data with the help of AI technology in teaching practice, make teaching decisions in line with AI theory and morality, and cooperate with AI to solve teaching problems, effectively improve students’ digital intelligence literacy and promote students’ development (Bitegeko et al., 2024). This differs significantly from teachers in other educational stages. First, the data types focus on micro-level teaching data in primary and secondary schools (such as student classroom engagement), rather than macro-level educational statistics or research data. Second, technology application must align with the cognitive development stages of primary and secondary school students. Third, competency goals serve dual purposes: enhancing teaching effectiveness and providing digital literacy foundational education for younger students. This necessitates integrating digital ethics education into daily instruction.

This study analyzes the intrinsic characteristics of teachers’ DAIC from five dimensions: its essence, attributes, composition, practice, and development. First, its essence manifests as a duality of methodology and specific competencies. On the one hand, the application of digital and intelligent technologies in education generates massive educational big data. Teachers’ data-driven instructional decisions embody DAIC, while Data-Driven Decision Making (DDDM) falls under the methodological category. This emphasizes teachers’ use of Database and Data Mining (DBDM) methods to design and implement instruction, thereby enhancing teaching effectiveness (Prenger and Schildkamp, 2018). On the other hand, teachers’ DAIC constitutes a capability framework encompassing digital knowledge, digital skills, and digital attitudes which teachers utilize to address teaching challenges encountered in the digital age. Second, its attributes emphasize digital-intelligent integration. Only when teachers cultivate an awareness of integrating digital and intelligent technologies, develop habits of data-driven thinking, and fully utilize AI to support data-driven instructional decisions can they advance effective teaching practices (Kim, 2024). Third, it is structured around higher-order thinking skills as a key element. In a digital-intelligent environment, teachers must possess a range of higher-order thinking abilities, including deeply analyzing and understanding the characteristics of digital-intelligent teaching, and critically and creatively using digital-intelligent methods and technologies in teaching practice (Lu et al., 2024). Fourth, in practice, it leans toward competency-based education. The target of teachers’ DAIC lies not only in guiding students to utilize digital and intelligent technologies to construct their own knowledge systems and develop their technical skills, but also in cultivating their higher-order abilities and competitiveness to better survive and thrive in an ever-changing era. This is precisely the competency required in the Data-Artificial Intelligent age (Ng et al., 2023). Finally, the goal is directed toward holistic development, serving not only students’ education but also teachers’ own developmental needs (Best, 2008). In the digital-intelligent society, society increasingly values whether students master higher-order abilities such as innovative thinking and collaborative communication. Additionally, data literacy, programming skills, critical reflection, digital ethics, and sound character are gradually gaining attention in the educational field (Huang et al., 2024). This shift represents an expansion of fundamental competency requirements for talent in the new era, enabling multidimensional student development supported by digital and intelligent technologies (Patrick et al., 2025). Thus, teachers’ DAIC encompasses not only knowledge and skills but also qualities related to thinking, personality traits, motivation, and attitudes which is a comprehensive, integrated, and advanced set of competencies.

### 2.2 Existing frameworks for teacher data and artificial intelligence literacy

Teachers’ DAIC, as a new concept representing the transformation of teacher literacy driven by AI technology in the new era, is essentially an optimization built upon existing foundations such as teacher data literacy and artificial intelligence literacy. Therefore, to ensure the analyzed literature is sufficiently representative, this study primarily draws upon existing research on these two types of models and discusses international experiences in teachers’ DAIC, laying the groundwork for subsequent research.

#### 2.2.1 Teacher data literacy model

The Teachers’ Data Literacy Model is a systematic conceptual and structural framework designed to define the multidimensional elements and their interrelationships required for teachers to achieve data-driven teaching. It encompasses the knowledge foundation, competency demonstration, attitudinal orientation, and ethical principles for teachers to identify, acquire, analyze, apply, and reflect on educational data within pedagogical contexts. Ultimately, it serves to optimize instructional decision-making, enhance student learning outcomes, and improve educational practices (Sandoval-Ríos et al., 2025). Reviewing existing research findings reveals four primary framework categories.

First is the DDDM framework. This concept has received significant attention from education authorities in countries such as the United Kingdom. Gummer and Mandinach (2015) developed a data literacy framework based on the evolution of teacher data literacy in the United States and the DDDM framework. It requires teachers to collect, analyze, and interpret all types of instructional data, transforming this data into actionable teaching decisions. Second is the Data-Driven Improvement Process Framework. Beck (2019) posited that the teacher data literacy competency framework encompasses ten indicators: acquiring data, analyzing data, understanding data, applying data, engaging in iterative inquiry using data, adjusting instructional strategies based on data, conducting personalized assessments using data, enhancing students’ data literacy, sharing data with peers, and utilizing data to guide teaching practices. Wolff et al. (2016) proposed that the structure of teacher data literacy comprises five components including problem identification, planning, data collection, analysis, and conclusion. Third is the Using Data Project Framework. Mandinach (2012) analyzed teacher data literacy across three levels, focusing on data-level users collecting and organizing data, information-level users analyzing and synthesizing information, and knowledge-level users integrating and prioritizing resource knowledge. Fourth is the Teacher Data Application Framework. Mandinach et al. (2015) expanded the original three dimensions into five dimensions which are specifying the problem, collecting and processing data, analyzing and interpreting data, translating data into teaching methods, and evaluating teaching methods.

Analysis of existing research on teachers’ data literacy reveals that previous studies have tended to emphasize teachers’ abilities to access, analyze, and process data, as well as the application of data in teaching processes. However, few scholars have addressed data ethics and the capacity to communicate using data.

#### 2.2.2 Teacher artificial intelligence literacy

Teachers’ AI Literacy is a comprehensive competency framework required for educators in the digital age to achieve deep integration of AI with teaching which centers on the organic unity of technological cognition, pedagogical application, and ethical stewardship (Sperling et al., 2024). The OECD (2025) defines it as the collection of abilities educators possess to understand, evaluate, and responsibly apply AI technologies to optimize teaching and learning, encompassing five dimensions including AI awareness, knowledge, application, thinking, and ethics (Chiu, 2025). Based on this, research analyzing AI-enhanced learning environments further indicates that this literacy must also incorporate the practical wisdom to combine the advantages of AI automation with the personalized needs of teaching (Umar et al., 2025). For instance, AI tools can simplify tasks like grading and lesson planning, allowing educators to focus instead on cultivating students’ critical thinking, creativity, and collaborative skills. Mills et al. (2024) further emphasized that the essence of teacher AI literacy lies not merely in technical operational skills, but in the higher-order ability to reconstruct teaching interaction models using AI as a medium. This requires both an awareness of AI’s limitations and guidance on digital ethics for students.

The widespread adoption of AI technology is driving a transformation in the teacher’s role from traditional knowledge transmitter to learning facilitator and AI collaborator. Central to this shift is unlocking the teacher’s creative potential and emotional engagement value (Chen et al., 2025). Teachers with high AI literacy can effectively leverage AI to analyze student learning patterns in real time (e.g., identifying weaknesses through adaptive platforms), shifting the focus of instruction from uniform lectures to personalized guidance. They become mentors on students’ learning journeys rather than one-way lecturers (Umar et al., 2025). Further research has confirmed that AI automation tools (such as intelligent lesson planning systems and automated grading software) can reduce teachers’ administrative workload by 40%, freeing them to devote more energy to designing engaging learning experiences (e.g., interdisciplinary projects, gamified lessons) and cultivating students’ emotional and social competencies through deep interaction (Yadav, 2025) especially important, they explicitly state that AI cannot replace teachers’ ability to build classroom communities through emotional resonance.

In summary, current research on teachers’ AI literacy primarily focuses on its conceptual framework and structural analysis, encompassing five dimensions. First is awareness-level literacy, such as actively learning about AI, rationally understanding AI, comprehending its technological advantages and risks, and regulating AI usage behaviors. Second is knowledge-level literacy, including fundamental AI concepts and characteristics, AI development history and trends, AI technical principles, and AI educational application scenarios. Third is AI thinking literacy, encompassing data literacy, computational thinking, and innovative thinking. Fourth is competency-based literacy, including human-machine collaboration skills, utilization of AI educational resources, operation of AI educational products, and development of AI educational applications. Fifth is AI ethical literacy, covering data privacy protection, cybersecurity, and AI resource interoperability and sharing.

#### 2.2.3 Teacher digital competency model

The European Union, Spain, Norway, and various organizations have released multiple “Digital Competence Frameworks for Teachers” tailored to different teaching requirements (García-Delgado et al., 2023; Norhagen et al., 2024; Suzer and Koc, 2024). Analysis of these frameworks reveals that they generally categorize the components of digital competence into three dimensions of knowledge, skills, and attitudes; the implementation of educational activities; and the roles teachers should assume in teaching and learning. The specific competency elements within these frameworks can be evaluated and measured through teachers’ observable behaviors. For instance, the European Framework for the Digital Competence of Educators categorizes components into professional engagement, digital resources, teaching and learning, assessment, empowering learners, and facilitating learners’ digital competence (Redecker and Punie, 2017).

It is evident that the frameworks proposed by various researchers and organizations lack dimensions related to the integration of data and artificial intelligence. Nowadays, AI-driven personalized data applications and intelligent technologies are sparking a new revolution, making the convergence of data and AI a defining feature of our era. In this era of Data-AI integration, isolated competencies or capabilities no longer meet societal demands for talent development. The concept of DAIC emerges as a response to the Data-AI era, representing essential future capabilities and qualities for educators. Consequently, constructing a teachers’ DAIC model has become an urgent priority.

### 2.3 Limitations of existing research

Existing teacher data and AI literacy frameworks predominantly exhibit a technology-oriented approach with distinct regional characteristics, yet they remain deficient in dimensional integration, cultural adaptation, and theoretical depth. From an international perspective, the European Union’s Dig Comp Edu takes digital skills refinement as its core strength, but its six-dimensional structure overemphasizes technical operations while lacking dimensions for AI ethics and personality traits (Caena and Redecker, 2019). The 2024 OECD Teacher AI Competency Framework explicitly emphasizes the ethical dimension, yet its internationally standardized design makes it difficult to adapt to regional contexts characterized by uneven distribution of educational resources between urban and rural areas (Bitegeko et al., 2024). Frameworks in East Asia, however, demonstrate a distinct localization orientation. For instance, a study from South Korea posits that teachers’ digital literacy comprises five components of understanding and applying technological tools and digital media, exploring and managing information and data, digital ethics and safety, relationship building, and teaching and assessment. It emphasizes digital collaborative relationships but fails to establish an organic integration logic for data and AI literacy, and lacks consideration for adaptability in low-resource environments (Park, 2023). Therefore, an important issue is how to integrate data, AI, and ethical elements while strengthening contextual relevance through distinctive indicators to address the dimensional fragmentation and cultural inadequacies of existing frameworks.

From a theoretical perspective, existing frameworks predominantly remain at the level of technological application, lacking in-depth explanations of the intrinsic logic underlying the integration of data and AI literacy. Based on critical theory of technology, the integration of data and AI literacy is not a simple superposition of skills (Heidegger, 1977). As the core raw material for AI, the value transformation of data relies on the rational application of AI tools; the separation of the two can easily lead to technological alienation. For instance, educators may blindly rely on AI decisions, losing their ability to judge data. In addition, existing frameworks generally overlook technology philosophy’s core emphasis on human-machine collaboration, failing to clearly distinguish between the teacher’s active constructive role and the auxiliary function of technological tools. Furthermore, while frameworks in East Asia focus on the attitude dimension, they do not systematically incorporate personality traits (such as proactivity and resilience in technology application) as independent dimensions, making it difficult to fully characterize the complete structure of teachers’ digital and AI literacy.

## 3 Methodology

### 3.1 Research design

First, this study employs literature review and comparative research methods to systematically examine and analyze the connotations, characteristics, and constituent elements of teacher data literacy, teacher AI literacy, and teacher digital competence. This approach clarifies the developmental trajectory and evolutionary path of teachers’ DAIC. By delineating the relationships among these various literacies/competencies, the study identifies the differences as well as the similarities or overlaps between them, laying the foundation for constructing a DAIC model. Subsequently, after extensively examining theoretical and practical research by international organizations and Western countries on advancing educational digitization and developing teachers’ ICT-related competencies (including teacher ICT competency standards and digital competency frameworks from organizations like UNESCO and the EU, as well as countries such as the United States, Norway, Spain, and Canada), the study analyzes the similarities and differences while deepening the understanding of trends in teachers’ DAIC development.

Second, the Delphi method is employed to iteratively refine the preliminary model, yielding the initial dimensions of the teachers’ DAIC model. Weight allocations are then assigned to each element of the revised model, resulting in a relatively comprehensive and well-structured framework and indicator system for primary and secondary school teachers’ DAIC.

Finally, a questionnaire survey method was employed to comprehensively assess the current state of teachers’ DAIC among primary and secondary school teachers. The survey targeted teachers and educational administrators from primary and secondary schools in eastern and central China. Based on the competency model and its secondary indicators, the “Survey Questionnaire on Teachers’ DAIC for Primary and Secondary School Teachers” was developed. This questionnaire underwent pilot testing and reliability/validity verification before being optimized through factor analysis.

### 3.2 Stage I of the research: framework construction

#### 3.2.1 Text analysis and expert selection

First, the text for this study was sourced from the September 2024 China Teacher Competency Needs Survey, which yielded 33,800 valid samples. Structural topic modeling (STM) was employed for data analysis, extracting 36 core themes such as smart tool application, data-driven decision-making, and ethical oversight. Second, the study invited 28 experts for consultation. All experts were engaged in teaching and research related to teacher education and educational informatization, with most holding the title of professor (researcher). Finally, among these 28 experts, 20 effectively participated in two rounds of expert consultation. Based on the primary and secondary components and secondary indicators described in the preliminary model of teachers’ DAIC, this study developed the “Expert Consultation Questionnaire on Components of Teachers’ DAIC” for use as the first-round expert consultation questionnaire. The information of experts is shown in the following Table 1.

**Table 1:** The information of experts.

Colleges	Number	Colleges	Number
Nanjing Normal University	4	Guangxi Normal University	1
South China Normal University	2	Hunan Normal University	1
Jiangsu Normal University	2	East China Normal University	1
Capital Normal University	2	Shaanxi Normal University	1
Wuhan University	2	Xizang Minzu University	1
Anhui Normal University	1	Zhejiang International Studies University	1
Beijing Institute of Education	1		

#### 3.2.2 Consultation process of the Delphi method

This study conducted the first round of expert consultation by emailing questionnaires to 28 preliminarily identified experts. Within the designated timeframe, feedback was received from 20 experts, all of whom completed the consultation questionnaire thoroughly, resulting in valid responses. Thus, the expert participation rate was 20/28 ≈ 0.714, indicating significant interest in the consultation topic of teachers’ DAIC and high engagement levels.

In Delphi-based research, the concentration of expert opinions can be represented by metrics such as the mean importance score (M_i_) and the frequency of maximum scores (K_i_) (Linstone and Turoff, 1975).


$$M_{i}=\frac{1}{m_{i}}\sum_{j=1}^{m}B_{\mathrm{ij}}$$

Where m_i_ denotes the number of experts participating in the evaluation of the i-th indicator, and B_ij_ represents the score assigned by the j-th expert to the i-th indicator. A higher value of M_i_ indicates greater importance of that indicator. The full-score frequency K_i_ denotes the ratio of experts who awarded full marks for a specific indicator to the total number of scoring experts. The value of K_i_ ranges between 0 and 1. A higher K_i_ indicates a larger proportion of experts awarding full marks to that indicator, signifying its greater importance.

Additionally, the concentration of expert consultation opinions can be analyzed using the mode, median, and the difference between the upper quartile (Q+) and lower quartile (Q−) (Q+ - Q−). Therefore, this study employed SPSS 26.0 and Excel 2019 to derive the concentration values for the first round of expert consultation opinions, with results presented in Table 2. According to the statistical results of expert opinions, among the 26 secondary indicators of teachers’ DAIC, 23 indicators had a mean (M) value of 4 or above, accounting for 88.5%. The remaining 3 secondary indicators had M values below 4 but above 3.5, representing 11.5%. This indicates that the average relative importance of all 26 secondary indicators exceeded 3.5 points, signifying their collective significance.

**Table 2:** Analysis of the concentration of expert opinions in the first round of consultation.

No.	Secondary indicators	M	K	mode	median	Q−	Q+	Q+ - Q−	Degree of concentration
1	D-AI integration awareness	4.45	0.65	5.00	5.00	3.75	5.00	1.25	<1.8
2	D-AI education philosophy	4.35	0.50	5.00	4.50	3.75	5.00	1.25	<1.8
3	D-AI attitude and values	4.60	0.65	5.00	5.00	4.00	5.00	1.00	<1.8
4	D-AI goal pursuit	4.30	0.50	5.00	4.50	3.75	5.00	1.25	<1.8
5	D-AI integration knowledge	3.65	0.55	5.00	4.50	2.25	5.00	2.75	>1.8
6	D-AI foundational knowledge	4.40	0.50	4.00	4.50	4.00	5.00	1.00	<1.8
7	D-AI teaching knowledge	4.45	0.65	5.00	5.00	3.75	5.00	1.25	<1.8
8	D-AI technical skills	4.45	0.55	5.00	4.50	4.00	5.00	1.00	<1.8
9	AI-driven data-based decision making	4.25	0.40	4.00	4.00	3.75	5.00	1.25	<1.8
10	Human-machine collaborative thinking	4.55	0.75	5.00	5.00	3.75	5.00	1.25	<1.8
11	Data-driven thinking	4.50	0.65	5.00	5.00	4.00	5.00	1.00	<1.8
12	Critical Thinking	4.70	0.80	5.00	5.00	4.75	5.00	0.25	<1.8
13	Creative Thinking	4.90	0.90	5.00	5.00	5.00	5.00	0.00	<1.8
14	Problem-based thinking	4.60	0.70	5.00	5.00	4.00	5.00	1.00	<1.8
15	D-AI learning and development	4.35	0.55	5.00	4.50	3.75	5.00	1.25	<1.8
16	D-AI teaching environment design	3.90	0.30	4.00	4.00	3.00	5.00	2.00	>1.8
17	D-AI teaching resource development	3.90	0.35	4.00	4.00	3.75	5.00	1.25	<1.8
18	D-AI teaching implementation	4.80	0.80	5.00	5.00	4.75	5.00	0.25	<1.8
19	D-AI teaching evaluation	4.70	0.70	5.00	5.00	4.00	5.00	1.00	<1.8
20	D-AI communication and collaboration	4.45	0.50	5.00	4.50	4.00	5.00	1.00	<1.8
21	D-AI education and empowerment	4.90	0.90	5.00	5.00	5.00	5.00	0.00	<1.8
22	Positivity	4.55	0.65	5.00	5.00	4.00	5.00	1.00	<1.8
23	Confidence	4.00	0.25	4.00	4.00	3.75	4.25	0.50	<1.8
24	Collaboration	4.00	0.50	5.00	4.00	3.00	5.00	2.00	>1.8
25	Responsibility	4.05	0.45	5.00	4.00	3.00	5.00	2.00	>1.8
26	Ethics	4.35	0.65	5.00	5.00	3.50	5.00	1.50	<1.8

D-AI: Data-Artificial Intelligent.

Based on the (Q+ - Q−) values, combined with specific expert feedback and relevant data, the components of teachers’ DAIC were screened and revised. This process led to the development of the second-round expert consultation questionnaire. It was distributed to the 20 experts who had completed the first-round questionnaire fully and validly. Within the designated timeframe, 20 valid expert feedback responses were received, resulting in a 100% participation rate for this round. This further demonstrates the high level of interest and engagement among these 20 experts regarding the topic of teachers’ DAIC. The analysis of the concentration of their opinions is presented in Table 3 below.

**Table 3:** Analysis of the concentration of expert opinions in the second round of consultation.

No.	Secondary indicators	M	K	Mode	Median	Q−	Q+	Q+ - Q−	Degree of concentration
1	D-AI technology affinity	4.55	0.60	5.00	5.00	4.00	5.00	1.00	<1.8
2	D-AI teaching awareness	4.65	0.65	5.00	5.00	4.00	5.00	1.00	<1.8
3	D-AI attitude and values	4.60	0.60	5.00	5.00	4.00	5.00	1.00	<1.8
4	D-AI objective pursuit	4.40	0.40	4.00	4.00	4.00	5.00	1.00	<1.8
5	D-AI foundational knowledge	4.70	0.75	5.00	5.00	4.25	5.00	0.75	<1.8
6	D-AI pedagogical knowledge	4.90	0.90	5.00	5.00	5.00	5.00	0.00	<1.8
7	D-AI technical skills	4.85	0.90	5.00	5.00	5.00	5.00	0.00	<1.8
8	Data-driven decision-making skills	4.20	0.35	4.00	4.00	4.00	5.00	1.00	<1.8
9	Human-machine collaborative thinking	4.55	0.70	5.00	5.00	4.00	5.00	1.00	<1.8
10	Critical thinking	4.60	0.70	5.00	5.00	4.00	5.00	1.00	<1.8
11	Creative thinking	4.55	0.70	5.00	5.00	4.00	5.00	1.00	<1.8
12	Problem-oriented thinking	4.60	0.75	5.00	5.00	4.25	5.00	0.75	<1.8
13	D-AI learning and development	4.70	0.70	5.00	5.00	4.00	5.00	1.00	<1.8
14	D-AI teaching environment application	4.55	0.55	5.00	5.00	4.00	5.00	1.00	<1.8
15	D-AI teaching resource integration	4.60	0.75	5.00	5.00	4.25	5.00	0.75	<1.8
16	D-AI teaching implementation	4.85	0.85	5.00	5.00	5.00	5.00	0.00	<1.8
17	D-AI teaching evaluation	4.70	0.70	5.00	5.00	4.00	5.00	1.00	<1.8
18	D-AI communication and collaboration	4.65	0.75	5.00	5.00	4.25	5.00	0.75	<1.8
19	D-AI education and empowerment	4.65	0.75	5.00	5.00	4.25	5.00	0.75	<1.8
20	Positivity	4.50	0.70	5.00	5.00	4.00	5.00	1.00	<1.8
21	Confidence	4.25	0.45	5.00	4.00	4.00	5.00	1.00	<1.8
22	Optimism	4.15	0.40	5.00	4.00	3.25	5.00	1.75	<1.8
23	Responsibility	4.30	0.40	4.00	4.00	4.00	5.00	1.00	<1.8
24	Ethics	4.75	0.80	5.00	5.00	5.00	5.00	0.00	<1.8
25	Achievement motivation	4.15	0.40	5.00	4.00	3.25	5.00	1.75	<1.8

D-AI, Data-Artificial Intelligent.

#### 3.2.3 Analysis of expert consultation results

The degree of agreement among expert comments is typically measured by the Kendall’s coefficient, which reflects the consistency of experts’ comments across all indicators (Abdi, 2007). Using SPSS 26.0 and nonparametric tests, the level of agreement among expert comments was determined, with results presented in Table 4.

**Table 4:** Kendall’s coefficient of expert agreement for the two rounds of expert consultation results.

Round	Kendall’s coefficient	X^2^	Experts’ number
First round	0.198	75.385**	20
Second round	0.552	209.819**	20

**p < 0 0.01.

As shown in Table 3, the Kendall’s coefficient for the first round of expert consultation was 0.198, with a *p*-value less than 0.01, indicating statistical significance. However, the relatively low Kendall’s coefficient suggests that the opinions of the 20 experts in the first round were divergent. Following revisions and adjustments to the components of teachers’ DAIC, the Kendall’s coefficient in the second round of expert consultation reached 0.552 which is much higher than the previous value, with a p-value less than 0.01. This indicates that the level of coordination among experts has now reached a high level, with strong consensus among the 20 experts and high reliability of the results. In summary, the 20 experts achieved a high level of coordination regarding the components of teachers’ DAIC, making the research findings acceptable. Therefore, the expert panel in this study meets the requirements of the Delphi method, demonstrating high expert authority and reliable consultation outcomes. This further validates the study’s reliability from a different perspective, confirming that the components of teachers’ DAIC are acceptable.

### 3.3 Stage I of the research: development and validation of measurement scales

#### 3.3.1 Initial scale

This study establishes a framework for teachers’ DAIC based on competency theory and Gagné’s Taxonomy of Learning Outcomes. After analyzing 165 relevant literature sources, a preliminary model of teachers’ DAIC was constructed using methods such as natural coding and word frequency statistics. After two rounds of iterative refinement, the final model comprises five primary indicators of D-AI awareness and concepts, D-AI knowledge and skills, D-AI higher-order thinking skills, D-AI teaching/learning application capabilities, and related personality traits, along with 25 secondary indicators. Based on this framework, an initial set of 50 items was developed using a 5-point Likert scale (1 = “Strongly Disagree,” 5 = “Strongly Agree”).

#### 3.3.2 Preliminary survey and item revision

The preliminary survey for this study targeted teachers from 9 schools (4 primary schools, 5 middle schools). A total of 254 questionnaires were distributed, with 215 valid responses collected (84.6% response rate). Through item analysis, two items with non-significant critical ratios (CR) (*p* > 0.05) were removed. Exploratory factor analysis (EFA) deleted four items with factor loadings <0.4. Additionally, two highly correlated items (*r* > 0.85) were merged based on item correlation analysis. Ultimately, 44 items were retained. Preliminary survey data indicated that the total scale Cronbach’s *α* = 0.983, with dimension-specific α coefficients ranging from 0.912 to 0.978. The KMO value was 0.966, and Bartlett’s test yielded χ^2^ = 7892.341 (*p* < 0.001), confirming suitability for EFA.

#### 3.3.3 Formal survey

The formal survey for this study employed stratified random sampling, covering 96 schools in total—46 primary schools and 50 junior high schools—with balanced representation across urban and rural areas as well as educational levels. A total of 2,346 questionnaires were distributed, with 2,052 valid responses collected (88.5% response rate). Among respondents, 1,128 were female (55.0%) and 924 were male (45.0%). By years of teaching, 423 respondents (20.6%) had less than 5 years of experience, 689 (33.6%) had 5–10 years, and 940 (45.8%) had over 10 years. By educational level, 845 (41.2%) taught at the primary level and 1,207 (58.82%) at the middle school level. By region, 1,082 respondents (52.7%) were from urban districts and 970 (47.3%) from townships. The distribution of demographic variables indicates a balanced and representative sample. Data quality control employed dual standards, excluding questionnaires completed in <180 s and invalid data where the same option was selected consecutively for 10 questions.

#### 3.3.4 Data analysis

This study used SPSS 26.0 for descriptive statistics, item analysis, and EFA. EFA utilized principal component analysis and maximum variance rotation, with factor structure determined based on eigenvalues >1, inflection points on the scree plot, and factor loadings >0.5. Confirmatory factor analysis (CFA) was conducted using Mplus 8.3 with maximum likelihood robust (MLR) estimation. Model fit indices were evaluated according to Hu and Bentler (1999) criteria: χ^2^/df < 3, CFI ≥ 0.90, TLI ≥ 0.90, RMSEA ≤ 0.08, and SRMR ≤ 0.08. Reliability was assessed using Cronbach’s *α* (internal consistency) and composite reliability (CR). Validity included aggregate validity (AVE ≥ 0.5), discriminant validity, and criterion-related validity.

## 4 Results

### 4.1 Framework for teachers’ DAIC

After text mining and expert consultation, this study finalized the teachers’ DAIC framework shown in Table 5, comprising five primary dimensions and 25 secondary dimensions. These components are considered essential qualities and abilities for teachers to effectively perform educational duties in the digital era. It is necessary to analyze and interpret these components to promote their understanding and effective application.

**Table 5:** Constituent elements and descriptions of teachers’ DAIC.

Primary indicator	Secondary indicators	Description
D-AI awareness and concepts	D-AI technology affinity	Possesses strong sensitivity to emerging technologies like AI, recognizes the impact of digital and intelligent technologies on individual and societal development, and acknowledges and supports their application.
	D-AI teaching awareness	Understands educational policies on digitalization and teaching demands in the digital era, recognizes the transformation of the teacher’s role and shifts in teaching methods and models, and demonstrates an exploratory mindset toward digital teaching.
	D-AI attitude and values	Maintains a balanced perspective on digital technologies, upholds technological rationality, and fully appreciates both the advantages and potential risks of digital technologies.
	D-AI objective pursuit	Possesses the mindset to leverage digital and intelligent technologies for professional growth and the vision to empower students’ autonomous development and social engagement through such technologies.
D-AI knowledge and skills	D-AI foundational knowledge	Understands the conceptual, theoretical, technical, tool-based, functional, and attribute-related knowledge of digital and intelligent technologies.
	D-AI pedagogical knowledge	Comprehends the relationship between digital and intelligent technologies and subject knowledge integration, instructional design, teaching models, and pedagogical strategies; possesses knowledge of teaching models such as virtual teaching, immersive teaching, human-machine collaborative teaching, and blended learning.
	D-AI technical skills	Master fundamental AI technologies and their application skills in daily life and teaching. Acquire competencies in data collection, cleaning, interpretation, and management. Possess skills in operating and maintaining digital intelligence teaching software, instructional equipment, learning platforms, and social media tools.
	Data-driven decision-making skills	Develop abilities to monitor, analyze, mine, and leverage data with AI assistance. Cultivate the capacity to transform data into information, convert information into pedagogical knowledge, and take action or adjust professional practice accordingly.
D-AI higher-order thinking skills	Human-machine collaborative thinking	Correctly understand human-machine relationships, fully recognize the strengths and limitations of both humans and intelligent machines, effectively allocate tasks between humans and machines, maintain human leadership, and make sound decisions in human-machine interactions.
	Critical thinking	Critically evaluate digital and intelligent technologies and their development, recognize AI drawbacks, critically assess machine recommendations, and conduct logical analysis, reasoning, judgment, and decision-making.
	Creative thinking	Proactively identify issues in digital-intelligent teaching, accurately apply digital-intelligent knowledge, analyze problems from novel perspectives, stimulate thinking, and propose innovative ideas and solutions.
	Problem-oriented thinking	Skilled at identifying problems from educational data and following the problem-solving approach: “Identify the problem → Understand the problem → Seek pathways → Determine solutions.”
D-AI teaching/learning application capabilities	D-AI learning and development	Utilize digital-intelligent technologies to support personal learning and development, address professional growth challenges through digital-intelligent teaching research activities, and achieve self-improvement.
	D-AI teaching environment application	Delivers personalized instruction in digital environments like smart campuses, intelligent classrooms, and virtual labs using IoT and cloud computing technologies.
	D-AI teaching resource integration	Integrates cross-disciplinary and cross-domain digital teaching resources, adapts and designs diverse digital teaching materials, and manages them effectively.
	D-AI teaching implementation	Selects appropriate digital teaching models based on instructional objectives, scientifically manages classroom activities with digital tools, and guides students in autonomous and collaborative learning using technology.
	D-AI teaching evaluation	Can utilize digital and intelligent assessment schemes and tools to conduct precise diagnosis and evaluation of courses and teaching, guide students to use digital and intelligent technologies for self-assessment and peer assessment, and propose targeted digital and intelligent teaching improvement plans based on assessment results.
	D-AI communication and collaboration	Can effectively use digital and intelligent social platforms and software to communicate and collaborate with colleagues, experts, students, and parents, and promote students’ digital and intelligent communication and collaboration.
	D-AI education and empowerment	Employ digital and intelligent technologies to deliver diverse holistic education (moral, intellectual, physical, aesthetic, and labor education), cultivate well-rounded personalities, and enhance students’ digital literacy and capabilities.
Related personality traits	Positivity	Demonstrate curiosity and enthusiasm for emerging technologies, proactively leveraging digital resources and tools to advance teaching and learning.
	Confidence	Possess confidence in mastering digital teaching and learning methodologies, leveraging technology to enhance instructional and learning outcomes.
	Optimism	Seeks guidance and adjusts promptly when encountering unfamiliarity with digital tools or teaching/learning challenges, maintaining an optimistic mindset.
	Responsibility	Participates in campus digitalization initiatives with a sense of responsibility to enhance educational quality and create social value through digital technologies.
	Ethics	Proactively complies with digital regulations, safeguards teaching data security, and prioritizes ethical oversight and guidance for students.
	Achievement motivation	Demonstrates strong motivation to achieve excellence in digital teaching and persists with unwavering dedication.

D-AI: Data-Artificial Intelligent.

As Table 5 clearly shows, nowadays teachers’ work is characterized by complexity and creativity, demanding a wide range of competencies and skills. With the rapid advancement of digital and intelligent technologies, their application in education has become increasingly widespread and profound. To a certain extent, this has brought convenience and efficiency to teachers’ work, freeing them from repetitive, mechanical tasks and allowing more time and energy for student management and teaching research. However, the integration of digital and intelligent technologies has not reduced the competencies teachers must possess. Compared to traditional teaching, the effective integration of these technologies with education requires teachers to develop more specialized, complex, and comprehensive competencies and qualities. Considering above, this study summarizes five key components based on Table 4. Detailed explanations follow.

First, D-AI awareness and concepts encompass four sub-dimensions, including D-AI technology affinity, D-AI teaching awareness, D-AI attitude and values, and D-AI objective pursuit. For instance, *Expert E4 noted, “Only by acknowledging the educational value of digital intelligence technologies will teachers proactively explore their application scenarios, which is a prerequisite for competency development.”*

Second, D-AI knowledge and skills encompass four sub-dimensions, including D-AI foundational knowledge, D-AI pedagogical knowledge, D-AI technical skills, and Data-driven decision-making skills. For instance, *Expert E9 emphasized, “Teachers must not only understand AI principles but also master the ‘data-to-instruction’ conversion logic, such as using AI to analyze homework data and adjust teaching strategies.”*

Third, D-AI higher-order thinking skills encompasses four sub-dimensions, including human-machine collaborative thinking, critical thinking, creative thinking, and problem-oriented thinking. For instance, *Expert E2 mentioned, “Human-machine collaboration does not mean machines replacing teachers, but rather division of labor under teacher leadership, requiring critical evaluation of the validity of machine suggestions.”*

Fourth, teachers’ D-AI teaching/learning application capabilities encompass seven sub-dimensions, including D-AI learning and development, D-AI teaching environment application, D-AI teaching resource integration, D-AI teaching implementation, D-AI teaching evaluation, D-AI communication and collaboration, and D-AI education and empowerment. For instance, *Expert E9 noted, “Teachers must act as leaders in the teaching process, adapting instruction to the specific teaching environment and employing diverse assessment methods to monitor effectiveness.”*

Finally, related personality traits encompass six sub-dimensions, including positivity, confidence, optimism, responsibility, ethics, and achievement motivation. For instance, *Expert E10 noted, “Resilience in facing technological challenges and a sense of responsibility toward data privacy protection are crucial safeguards for DAIC.”*

### 4.2 Scale validation

#### 4.2.1 Exploratory factor analysis

**Figure 1:**
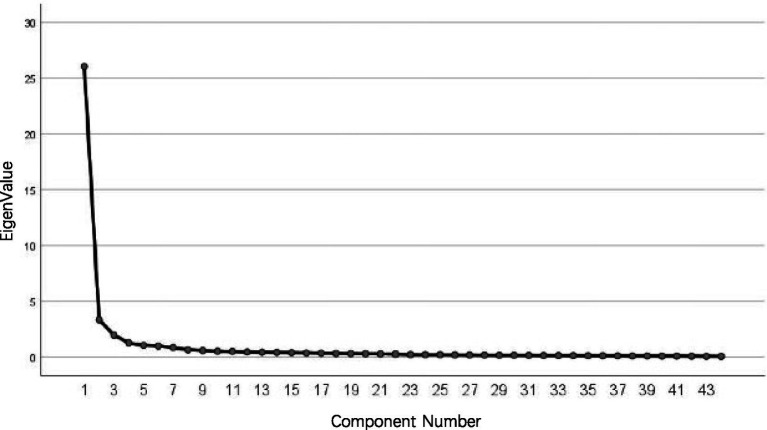
Gravel chart.

This study first employed EFA to analyze 215 pre-survey data. Gravel chart analysis revealed that the scale could extract five common factors, a result that fully aligned with the theoretical framework dimensions, as illustrated in Figure 1.

Based on the factor loading analysis shown in Table 6, this study further reveals that the cumulative variance explained by the final five factors reached 76.397%. Among the five extracted common factors, all 44 items exhibited factor loadings exceeding 0.50, meeting standard statistical requirements. Factor one, including 14 items (Q25–Q38), corresponds to teachers’ D-AI teaching/learning application capabilities. Factor Two, including 6 items (Q39–Q44), corresponds to related personality traits. Factor 3 including 10 items (Q9–Q18) corresponds to D-AI knowledge and skills. Factor 4 including 8 items (Q1–Q8) corresponds to D-AI awareness and concepts. Factor 5 including 6 items (Q19–Q24) corresponds to D-AI higher-order thinking skills. This indicates the scale possesses very high construct validity. Furthermore, factor loading analysis of all items reveals that all loadings fall between 0.512 and 0.867, meeting the criterion of >0.5.

**Table 6:** Results of exploratory factor loadings analysis.

Items	Factors				
	1	2	3	4	5
Q36	0.841				
Q34	0.829				
Q33	0.811				
Q35	0.801				
Q38	0.782				
Q32	0.761				
Q31	0.755				
Q30	0.752				
Q29	0.75				
Q37	0.739				
Q28	0.685				
Q27	0.604				
Q26	0.531				
Q25	0.512				
Q40		0.847			
Q41		0.844			
Q39		0.811			
Q42		0.767			
Q43		0.63			
Q44		0.617			
Q13			0.833		
Q16			0.794		
Q12			0.788		
Q9			0.771		
Q14			0.664		
Q11			0.605		
Q10			0.585		
Q17			0.574		
Q15			0.556		
Q18			0.546		
Q2				0.867	
Q4				0.828	
Q3				0.817	
Q1				0.784	
Q8				0.706	
Q7				0.682	
Q5				0.615	
Q6				0.610	
Q20					0.71
Q19					0.684
Q21					0.655
Q24					0.610
Q23					0.592
Q22					0.569
Percentage of variance	59.157	7.559	4.461	2.873	2.347
Cumulative percentage	59.157	66.716	71.177	74.05	76.397

#### 4.2.2 Confirmatory factor analysis

Following EFA of the scale, this study conducted CFA on 2,052 formal survey data to ensure the scale’s reliability. Analysis results showed, χ^2^/df = 3.11, CFI = 0.938, TLI = 0.931, RMSEA = 0.046 (90% CI: 0.043–0.049), SRMR = 0.040, with all indices meeting ideal standards. Standardized factor loadings ranged from 0.512 to 0.867, with 80% of items exhibiting loadings >0.6. This indicates strong item-dimension associations and model stability, confirming the scale’s excellent model fit.

#### 4.2.3 Reliability and validity analysis

To further ensure the applicability of this scale in future research, this study conducted reliability and validity tests on the formal data. Reliability analysis revealed a total Cronbach’s *α* of 0.983, with dimension-specific α coefficients as follows, D-AI awareness and concepts (0.912), D-AI knowledge and skills (0.935), D-AI higher-order thinking skills (0.928), D-AI teaching/learning application capabilities (0.978), and related personality traits (0.915), with all coefficients exceeding 0.90. Additionally, the composite reliability (CR) for each dimension ranged from 0.915 to 0.979, all surpassing the baseline requirement of 0.70, indicating excellent internal consistency of the scale. Validity analysis revealed that the average variance extracted (AVE) for each dimension ranged from 0.523 to 0.687, all exceeding 0.50, indicating good convergent validity. Furthermore, the square roots of the AVE values (0.723–0.829) for each dimension were all greater than the correlation coefficients between that dimension and other dimensions (0.527–0.605), meeting the criteria for discriminant validity. Regarding criterion-related validity, the scale’s total score showed a significant positive correlation with teachers’ information-based teaching competency scores (r = 0.472, *p* < 0.01) and with digital teaching satisfaction scores (r = 0.455, *p* < 0.01), indicating strong criterion validity.

## 5 Conclusion and discussion

### 5.1 Conclusion

This study employs a mixed-methods approach to systematically construct a framework for teachers’ DAIC and validate its measurement scale, offering a novel perspective to existing research.

First, this study clarifies the core dimensions and structural framework of teachers’ DAIC. The constructed framework comprises five primary dimensions, including D-AI awareness and concepts, D-AI knowledge and skills, D-AI higher-order thinking skills, D-AI teaching/learning application capabilities, and related personality traits, along with 25 sub-dimensions. This forms a complete logical chain of “cognition (awareness - knowledge) - capability (thinking - application) - personality.” Among these, D-AI teaching/learning application capabilities (weight 0.318) serves as the core practical vehicle, directly reflecting the integration effectiveness of DAIC technologies with teaching. D-AI higher-order thinking skills (weight 0.187) acts as the key core, determining teachers’ depth of mastery and innovative application level of digital technologies. Both of them together form the core pillars of teachers’ DAIC.

Second, standardized scales with high psychometric quality were developed and validated. The final 44-item Scale of Teachers’ DAIC demonstrated excellent reliability and validity. The total Cronbach’s *α* coefficient reached 0.983, with all dimension α coefficients exceeding 0.90. CR ranged from 0.915 to 0.979, indicating excellent internal consistency. CFA revealed good model fit (χ^2^/df = 3.11, CFI = 0.938, TLI = 0.931, RMSEA = 0.046, SRMR = 0.040). All standardized factor loadings exceeded 0.5, meeting criteria for aggregate validity (AVE > 0.5) and discriminant validity. This establishes the scale as a standardized instrument for assessing teachers’ DAIC.

### 5.2 Discussion

#### 5.2.1 Theoretical contribution: triple integration overcomes existing research limitations

The Data - Artificial Intelligent Competence framework and scale developed in this study achieve a triple theoretical integration, addressing gaps in existing research. First, it represents a systematic breakthrough in dimensional integration. It overcomes the fragmented limitations of existing research that treats data literacy, AI literacy, and digital competence as separate entities. For the first time, it systematically integrates the three core elements of data intelligence - AI collaboration - ethical oversight, clearly establishing the central role of new factors in the digital intelligence era, such as digital ethics oversight and human-machine collaborative decision-making. This integration not only addresses the shortcomings of the EU Dig Comp Edu framework (Redecker and Punie, 2017), which emphasized digital skills while weakening the ethical dimension, but also aligns with the core viewpoint of UNESCO’s Teacher AI Literacy Framework that ethical responsibility is a core competency, thereby refining the theoretical system of teachers’ DAIC in the digital intelligence era. Second, it integrates theoretical and practical perspectives. Through text mining of competency demands from 33,800 frontline teachers, combined with Delphi consultations from 28 international experts, it achieves an organic unity of top-down theoretical deduction and bottom-up practical induction. The framework’s application of digital-intelligent teaching environments, encompassing virtual experimental teaching and interdisciplinary resource integration, directly addresses primary and secondary teachers’ practical demands for digital-intelligent technologies in subject instruction. This avoids the disconnect of traditional frameworks that overemphasize theory while neglecting practice, enhancing the theory’s practical explanatory power. Third, comprehensive coverage of explicit and implicit competencies. Innovatively, personality traits (such as positivity, responsibility, and ethics) are incorporated as independent dimensions within the system. This breaks through the superficial perspective of most studies that focus solely on explicit competencies like technical skills and knowledge application. It resonates with the core principle of competency theory that implicit traits are the core of high performance (McClelland, 1973). This design enables the framework to evaluate not only teachers’ technical application abilities but also professional competencies in the digital and intelligent era, achieving a comprehensive portrayal of teachers’ DAIC.

In addition, the scale demonstrates significant advantages in localized adaptation. Compared to localized versions of the EU Dig Comp Edu scale, this scale’s items closely align with Chinese primary and secondary school teaching contexts. Items such as urban–rural resource allocation, data privacy protection, and participation in campus digital and intelligent infrastructure development specifically address shortcomings in cultural adaptation and contextual applicability found in international scales. This provides a more precise tool for assessing teachers’ DAIC within the Chinese educational context.

#### 5.2.2 Practical implications: precisely empowering teacher development and educational management

Based on framework dimension weights and regional differences, teacher training should adopt a modular design combining common foundational elements with personalized enhancement. Common modules focus on foundational dimensions such as digital intelligence awareness and concepts, as well as digital intelligence knowledge and skills. Online courses are used to disseminate basic data knowledge and intelligent tool operations. Customized modules should precisely address regional needs, such as offering advanced courses like AI instructional innovation design or interdisciplinary digital-intelligence project development for eastern educators, while providing targeted training in foundational data tool applications and digital-physical integration teaching for central, western, and rural teachers. Additionally, supporting resources should include AI teaching toolkits (containing open-source offline data processing software and localized AI lesson planning templates), 5-min scenario-based micro-training (e.g., rapid AI courseware generation), and school-based technical mutual aid groups (where school technical lead teachers mentor peers to resolve real-time operational challenges). This approach addresses rural teachers’ information silos and disconnect between digital literacy and teaching practice. Training methods must emphasize practical application through simulated virtual teaching scenarios, school-based action research, and case studies of AI-assisted student assessment diagnostics. This enhances teachers’ human-machine collaboration thinking and digital intelligence application skills while cultivating their core competency to critically evaluate machine recommendations.

In teacher evaluation and recruitment, this scale can serve as a standardized tool. Regionally tailored adjustments to dimension weights can be made—for instance, rural schools may prioritize dimensions like digital-physical integration, responsibility ethics, and low-threshold tool operation to precisely identify competency gaps. Education authorities can use assessment outcomes to construct regional DAIC profiles and formulate differentiated policies. Increase training funding and technical support for underdeveloped areas (e.g., distributing offline digital teaching toolkits, establishing regional resource-sharing platforms) to promote equitable distribution of digital educational resources. Simultaneously, establish an integrated monitoring system linking teachers’ DAIC development to student digital literacy enhancement and instructional quality improvement. This creates a closed-loop management mechanism of assessment-diagnosis-training-reassessment, enabling synergistic advancement of teacher professional development and educational quality enhancement.

## 6 Limitations

This study has limitations in four areas that warrant further refinement. First, the representativeness of the sample is constrained. While the formal survey sample covers primary and secondary school teachers in eastern and central China, it excludes underdeveloped regions in the central and western parts of the country, ethnic minority areas, and rural schools with weaker resource endowments. Additionally, the sample exhibits an imbalance in the proportion of urban versus rural teachers. This geographical concentration and group homogeneity may limit the generalizability of findings nationwide. It fails to fully reflect the actual teachers’ DAIC levels in diverse regions (e.g., remote western counties) or resource conditions (e.g., small rural schools), nor does it adequately capture the specific needs of teachers in bilingual education settings within ethnic regions. Second, there are limitations in data type. This study employs cross-sectional data for analysis, capturing only the static characteristics of teachers’ DAIC at a specific point in time. It cannot track their dynamic development trajectories or long-term evolution patterns in response to training interventions, policy adjustments, or technological iterations (e.g., changes in competency dimensions after one year of DAIC training). Furthermore, cross-sectional data struggles to eliminate confounding variables (such as school investment in information technology), making it impossible to establish causal links between training interventions and competency improvements or to reveal the temporal mechanisms influencing competency development. Third, there is a lack of cross-cultural validity testing. The scale’s development and validation were based solely on China’s K-12 educational context, without conducting adaptability tests across different cultural backgrounds or educational systems (e.g., Belt and Road countries, Western primary education systems, Southeast Asian bilingual education settings). The cross-cultural universality of its measurement dimensions and item wording remains unverified, limiting both the scale’s international applicability and its capacity to support comparative studies of teachers’ DAIC across diverse cultural contexts. Fourth, control of common method bias is inadequate. The core data relies on teacher self-report questionnaires. Although validity tests were conducted, no triangulation with multi-source data (e.g., classroom digital teaching observation records, student digital literacy assessment results, school digital training archives) was performed. Potential social desirability bias in subjective reports (e.g., teachers overestimating their digital ethics awareness) may still influence measurement outcomes across certain dimensions.

To address the aforementioned limitations, this study suggests that future research could advance in the following four areas. First, expand sample coverage and regional comparisons. Broaden the geographic and demographic scope of the sample to include teachers from underdeveloped regions in central and western China, ethnic minority areas, and rural schools. Through regional comparative analysis, delve deeper into the developmental disparities in teachers’ DAIC across different regions, and identify regionally adaptable pathways for enhancing DAIC. Second, implement longitudinal tracking and causal inference. Employ longitudinal research designs to conduct 1–2 year follow-ups with teachers, systematically examining the long-term trajectory of teachers’ DAIC development and the mechanisms through which key factors, such as training interventions, policy support, and school organizational environments, exert influence. This will provide empirical evidence for establishing causal relationships. Third, advance cross-cultural validation and adaptation. Translate the scale into multiple languages and conduct empirical testing across educational systems in Belt and Road countries and diverse cultural contexts. This will reveal cultural commonalities and specificities in teachers’ DAIC, optimize the scale’s cross-cultural adaptability, and provide a comparable tool for international research on teachers’ DAIC. Fourth, design intervention experiments and validate outcomes. Based on the DAIC framework developed in this study, create targeted training intervention programs. Employ pre- and post-test designs comparing experimental and control groups to empirically verify the effectiveness and feasibility of these training programs, providing more direct and actionable practical evidence for enhancing teachers’ DAIC.

## Data Availability

The raw data supporting the conclusions of this article will be made available by the authors, without undue reservation.
